# Gender differences in exposure to SRH information and risky sexual debut among poor Myanmar youths

**DOI:** 10.1186/1471-2458-13-1122

**Published:** 2013-12-05

**Authors:** Phyu Phyu Thin Zaw, Tippawan Liabsuetrakul, Edward McNeil, Thien Thien Htay

**Affiliations:** 1 Department of Medical Research (Upper Myanmar), Pyin Oo Lwin, Myanmar; 2 Epidemiology Unit, Faculty of Medicine, Prince of Songkla University, Hat Yai, Songkhla 90110, Thailand; 3 Ministry of Health, Nay Pyi Taw, Myanmar

**Keywords:** Youths, Gender difference, SRH information, Risky sexual debut

## Abstract

**Background:**

Globally, the proportion of youths has been steadily increasing, especially in Asia. This vulnerable population has limited exposure to sexual and reproductive health (SRH) information leading to various reproductive health (RH) problems including risky sexual debut, unwanted pregnancy, unsafe abortion as well as STI/HIV infections. Among known social variations which influence youth’s RH, gender differences are critical for planning necessary gender appropriate interventions. This study aimed to identify gender differences in exposure to SRH information and risky sexual debut as well as associated factors among Myanmar youths in poor suburban communities of Mandalay City.

**Methods:**

A total of 444 randomly selected youths (aged 15–24 years) from all poor, suburban communities in Mandalay City took part in our survey. Gender differences in exposure to SRH information and risky sexual debut were assessed by bivariate analysis. Multivariate logistic regression was used to confirm gender differences and identify independent factors associated with main outcomes separately for males and females as well as for both.

**Results:**

Of 444 youths interviewed, 215 were males and 229 were females. Gender differences were seen in both exposures to SRH information (p = 0.013) and risky sexual debut (p = 0.003). These gender differences were confirmed by multivariate analysis even after adjusting for other risk factors. For exposure to SRH information, only age group and schooling status were significant factors for females. As well as those two factors, media exposure and parental guardianship were significant factors among males. Only positive norm of premarital sex increased the likelihood of risky sexual debut among males. In contrast, unwillingness at sexual debut was a risk factor and a higher education level was a protective factor for risky sexual debut among females.

**Conclusions:**

Limited exposure to SRH information and high risky sexual debut among poor youths were found. There were different influential factors for RH behaviors between males and females. Policy makers as well as local RH care providers should be aware of these differences. Dissemination of reliable SRH information among youths through possible mass media, especially among males, is an urgent issue.

## Background

Globally, the proportion of youths has been steadily increasing, especially in developing countries across Asia. Among all youths, 60% is currently living in Asia [1]. The period of adolescence or young adulthood is important since it is a transitional period in which youths start to become curious about their sexuality and experiment with new sexual activities. It is also an opportunistic period for them in which they must learn to adapt and make important decisions for embracing their future. Failure to provide adequate sexual and reproductive health (SRH) information to youths combined with their lack of exposure to this information during this transitional period may lead to major public health implications such as STI/HIV infections, unwanted pregnancies and unsafe abortions as well as risky sexual debut [2,3].

First sexual intercourse, or sexual debut, is a normal part of human development when youths are exposed to sex for the first time in their lives. Sexual debut is important because the earlier age at sexual debut, the more risky sexual behaviors become later in life [4]. According to United States’ Centers for Disease Control and Prevention, risky sexual behaviors include having sex at an early age (16 years or younger), having multiple sexual partners or non-regular partners, having sex while under the influence of alcohol or drugs, and unprotected sexual behaviors (not using any condoms or contraceptives). Nowadays, early and risky sexual debut among youths is increasing worldwide along with higher adverse RH consequences [5]. Adequate exposure to proper SRH information has been shown to have a protective effect on risky sexual behaviors and reduce negative RH outcomes [6,7]. However, various socio-economic factors deter youths from receiving basic SRH information as well as achieving a safe and responsible sexual debut [3,5,8,9].

Among social variations which influence youth’s RH behaviors, gender difference is an important issue to focus on nowadays. Evidence has shown that males take more sexual risks than females [5,9,10]. Younger age at first sex and premarital sex is more common among males [10,11]. Females have more negative attitudes towards sex, such as shame and guilt, compared to males and are less likely to engage in premarital sex as well as sex with non-regular partners [9]. Females are socio-economically more vulnerable, especially in poor communities and are more likely to be coerced into sexual debut and early marriage than males [12,13]. In addition, SRH for youths is a culturally sensitive issue in most Asian countries [14]. This issue is inadequately addressed in some countries leading them to have more adverse RH outcomes. A thorough identification of gender specific factors which influence exposure to SRH information and risky sexual debut among youths will help to establish culture and gender appropriate solutions in improving youth’s RH in most Asian countries.

Myanmar is one of the Asian countries in which youths constitute a significant proportion of the country’s population [15]. Early, premarital and risky sexual debut is not uncommon [16-18]; however issues on youth’s sexual behaviors have so far been poorly understood and scarcely addressed in the country due to cultural reasons. Determining the factors associated with exposure to SRH information and risky sexual debut among youths is an urgent issue for policy makers. This study thus aimed to identify gender differences in exposure to SRH information and risky sexual debut and their associated factors among poor Myanmar youths in suburban communities of Mandalay City.

## Methods

### Study area

Myanmar is located in South-East Asia, bordered by India, Bangladesh, China, Thailand and Laos. Mandalay City, situated in central Myanmar, is a densely populated area with a relatively low cultural, racial and social heterogeneity. There are 10 poor communities situated in suburban areas of the city which are enriched with hard-to-reach, informal settings and people with a low socio-economic status.

### Study design and participants

This sub-study is a part of a larger community-based, cross-sectional study with three main objectives, namely, to assess the levels of accessibility to and utilization of RH services, to explore their associated factors including youth’s risky sexual debut and to find out ways to improve youth’s access to and use of RH services. In the larger study, youths as well as RH care providers (governmental, non-governmental and local drug-shop owners) and community leaders were included (participant triangulation) by using both quantitative and qualitative methods (method triangulation) [19]. In this sub-study, only male and female youths aged between 15–24 years residing in 10 poor, suburban communities of Mandalay City were included in order to explore the gender differences in exposure to SRH information and risky sexual debut among poor youths. The sample size of the main study was sufficient to test the hypothesis. We assumed a prevalence of risky sexual debut among males of 50% (no previous data). The study sample of 430 (215 females and 215 males), would have 80% power to detect a difference of about 15% between males and females (odds ratio of 1.4) in risky sexual debut with 95% confidence.

### Sampling methods

The process of sampling was conducted using the following steps.

1. Since the structure of Mandalay City is composed of rectangular blocks, the suburban areas can readily be identified. There are four broad suburban areas on the outskirts of Mandalay City.

2. In those suburban areas, there were a total of 10 poor communities (identified by township medical officers and municipal authorities based on their socio-economic status and RH outcomes). All these poor communities were included in our study.

3. Each community had a community leader as well as volunteers who were assigned as the leaders of clusters of 10 households. Assigning a leader for every 10 households in a community is a traditional way of community management in almost all Myanmar communities.

4. We asked the community leaders to identify youths aged 15–24 years (a standard definition by World Health Organization) in their communities. We suggested them to instruct household cluster leaders (1 cluster = 10 households) to check the youth’s ID card to verify their dates of birth. If the youth did not have any identification, their traditional birth record was used instead.

5. The household cluster leaders gave the community leaders the lists of youths stratified by sex living in their responsible cluster. The community leaders checked the lists and combined them and handed them to our research team. The whole process was supervised by municipal authorities as well as local midwives.

6. The total number of youths in each community given by the community leaders to our research team ranged from 300–350. Altogether, a total of 3,263 youths were identified living in those 10 communities of Mandalay City.

7. After obtaining the final lists, a roughly equal number of males and females were randomly selected from each community using a random number generator based on the sex-stratified lists.

### Outcome measures

The outcome measures were high exposure to SRH information and risky sexual debut. Youths who ever attended the SRH information classes at least once in their lifetime or had a high knowledge on “sexuality” or “sexually transmitted infections/human immunodeficiency virus infection (STI/HIV)” were defined as having a high exposure to SRH information. SRH information classes included any health education sessions/talks/classes that emphasized on SRH given at schools, clinics, or other places including peer-based education and excluding mass media. Knowledge of STI/HIV was evaluated by asking youths if they knew the common causes of STI or HIV, their modes of transmissions and ways of prevention. The maximum score possible was 16. Knowledge on sexuality was measured by asking for knowledge of any signs of puberty for both boys and girls, methods to avoid pregnancy and danger-periods during the menstrual cycle. The maximum score possible was 17. Having a high knowledge on both “STI/HIV” and “sexuality” was defined as youths having knowledge scores which were higher than the respective means (8 for the knowledge of STI/HIV and 8.5 for the knowledge of sexuality). Risky sexual debut in our study was defined as youths who had an early sexual debut (at the age of 16 or younger) or a premarital sexual debut or a sexual debut with a non-regular partner or without using any condoms/contraceptives. Sexual debut was defined as sexual intercourse (involving vaginal or anal penetration) for the first time. Non-regular partner was defined as any partner outside of marriage or regular union (i.e. not including husband/wife or boyfriend/girlfriend but including commercial sex worker or stranger).

#### Independent variables

Independent variables included demographic and socioeconomic characteristics, namely age group (late adolescents: 15–19 years and young adults: 20–24 years), gender, schooling status (student or out-of-school), level of education, occupation, personal income, sexual exposure, marital status, self-rated access to condoms/contraceptives, unwillingness to have sex at sexual debut, exposure to mass media on SRH (never versus ever in their life), sources of SRH information (reliable or unreliable), norm of premarital sex, and type of guardianship (parent or non-parent). The level of education was classified as low (middle school and below) or high (high school and above). The international poverty line of US$ 37.5 or 30,000 kyat (800 kyat per US$ 1) was regarded as the threshold for a low personal income per month. Youths who knew where to get condoms/contraceptives and said that it was easy to get them were defined as youths having a high self-rated access to condoms/contraceptives and low otherwise. Unwillingness to have sex at sexual debut was defined as youths who were forced to have sex or those who had to have sex not for pleasure but for money to survive at their first intercourse. Exposure to mass media on SRH was defined as youths who replied that they got information on sexuality, STI/HIV, contraceptives or maternal health and pregnancy through any kind of mass media such as books, television, radio, or the Internet. Source of SRH information was classified into reliable (health personnel) or unreliable (friends, older youths etc.). If a youth responded that his/her friends were having premarital sex and it was very common and usual in his or her community and perceived that there was nothing wrong with premarital sex, then the perceived norm of premarital sex was classified as “positive”.

### Statistical analysis

Differences in exposures to SRH information and risky sexual debut were initially assessed by bivariate analysis. Variables which had a *p*-value of less than 0.2 were included in the multivariate logistic regression analysis. Independent variables associated with the main outcomes were also assessed separately for males and females as well as for both. Variables having a *p-*value less than 0.05 in the final model were considered as statistically significant.

### Ethical considerations

The study was approved by the Institutional Ethics Committee of the Faculty of Medicine, Prince of Songkla University, Hat Yai, Thailand and the Ethics Committee of the Department of Medical Research (Upper Myanmar) before the study was conducted. All proposed youths had a right to participate in the study using informed consent. Their anonymity and confidentiality was stringently maintained throughout the study.

## Results

### Socio-demographic characteristics

Of 444 youths approached, 215 males and 229 females agreed to participate, giving a response rate of 100%. Table 1 shows a comparison of demographic characteristics between males and females. There were no differences in age or schooling status. However, level of education was significantly different and females were more likely to be unemployed and have a lower personal income. Females were also more likely to be married and exposed to sex. Self-rated access to condoms/contraceptive was significantly higher among males. The proportion of youths who had their first sexual debut unwillingly or were forced to have sex at their sexual debut was higher among females (26% vs 8%). Males had a higher exposure to mass media (42% vs 23%) but less exposure to reliable sources of SRH information. Females were more likely to have a negative perceived norm of premarital sex and more likely to be under the guardianship of their parents.

**Table 1:** Comparison of demographic characteristics between male and female youths

	**Male**	**Female**	
**Factor**	**215**	**229**	**P value**
	**n (%)**	**n (%)**	
Age group			0.284
Adolescent	118 (54.9)	113 (49.3)	
Young adult	97 (45.1)	116 (50.7)	
Schooling status			0.101
Student	20 (9.3)	34 (14.8)	
Out-of-school	195 (90.7)	195 (85.2)	
Education level			0.013
Low	129 (60)	164 (71.6)	
High	86 (40)	65 (28.4)	
Occupation			< 0.001
Unemployed	69 (32.1)	126 (55)	
Employed	146 (67.9)	103 (45)	
Personal income per month			< 0.001
≤30,000	52 (24.2)	123 (53.7)	
>30,000	163 (75.8)	106 (46.3)	
Sexual exposure			0.011
Never exposed	115 (53.5)	94 (41)	
Ever exposed	100 (46.5)	135 (59)	
Marital status			< 0.001
Never married	150 (69.8)	100 (43.7)	
Ever married	65 (30.2)	129 (56.3)	
Self-rated access to condoms/contraceptives			
Low	76 (35.3)	118 (51.5)	< 0.001
High	139 (64.7)	111 (48.5)	
Willingness to have sex at sexual debut			< 0.001
Willing	92 (92.0)	100 (74.1)	
Unwilling	8 (8.0)	35 (25.9)	
Media exposure to SRH			< 0.001
Never	125 (58.1)	177 (77.3)	
Ever	90 (41.9)	52 (22.7)	
Source of SRH information			0.004
Unreliable (not health personnel)	86 (40)	61 (26.6)	
Reliable (health personnel)	129 (60)	168 (73.4)	
Norm of premarital sex			0.013
Positive	87 (40.5)	66 (28.8)	
Negative	128 (59.5)	163 (71.2)	
Guardianship			0.01
Parent	123 (57.2)	159 (69.4)	
Other	92 (42.8)	70 (30.6)	

### Gender difference in exposure to SRH information and other associated factors

The overall levels of exposure to SRH information among male and female youths in our study were 61.9% and 73.4%, respectively. Table 2 compares the exposures to SRH information between males and females. There were no differences in attending SRH education classes and knowledge of sexuality. However, females had a higher knowledge of STI/HIV resulting in a higher overall exposure to SRH information (*p*-value = 0.013).

**Table 2:** Comparison of high exposure to SRH information between males and female youths

	**Males (n = 215)**	**Females (n = 229)**	**P value**
**Factor**	**n (%)**	**n (%)**	
High knowledge of STI/HIV	108 (50.2)	153 (66.8)	< 0.001
High knowledge of sexuality	13 (6.0)	8 (3.5)	0.08
Ever attended SRH information classes	67 (31.2)	80 (34.9)	0.5
**High exposure to SRH information**	**133 (61.9)**	**168 (73.4)**	**0.01**

**Figure 1:**
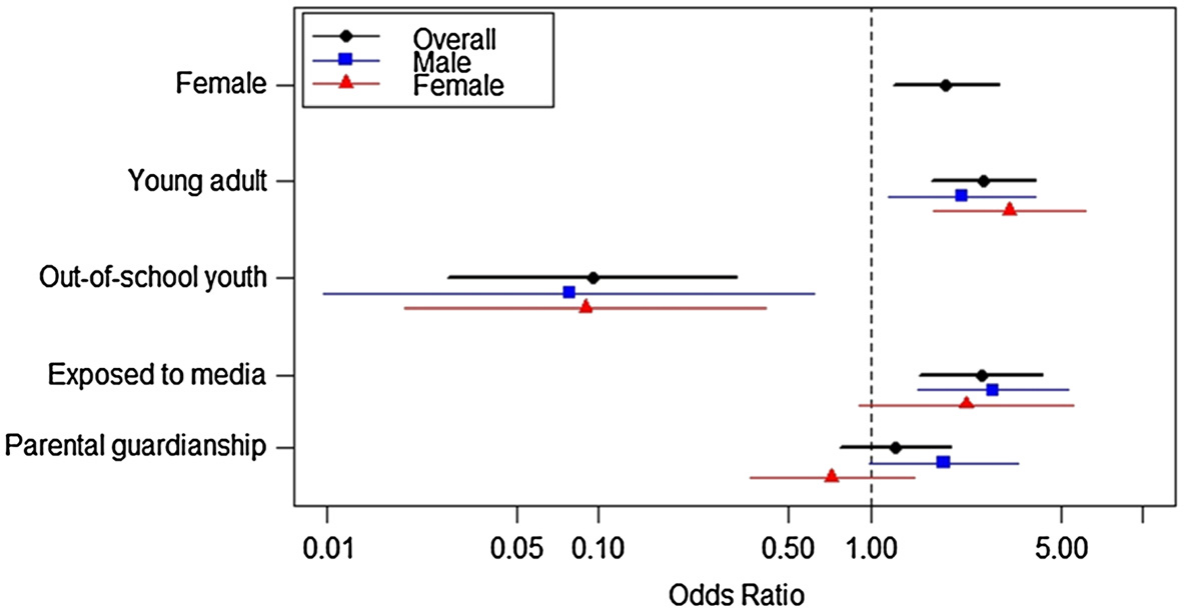
Overall and sex stratified factors associated with exposure to SRH information.

Figure 1 compares factors associated with exposure to SRH information from the multivariate analysis. Among all youths, being an adolescent, out-of-school and never being exposed to mass media on SRH were factors that were associated with having a lower likelihood of exposure to SRH information. Among females, being an adolescent and out-of-school were the only significant risk factors for lower exposure to SRH information. Among males, media exposure and parental guardianship were protective factors while being an adolescent and out of school were risk factors.

### Gender difference in exposure to risky sexual debut and other associated factors

Table 3 shows a comparison of risky sexual behaviors at sexual debut between males and females. There were no differences in age or use of condoms/contraceptives at sexual debut between males and females. Similar proportions of males and females were exposed to early sexual debut before they turned 17. While 35% of males had premarital sexual debut, only 4% of females had that behavior. Rate of non-use of contraceptives/condoms at sexual debut was 52% among males and 47% among females. The behavior of having a sexual debut with non-regular partner was higher among males (27%) while this behavior was not common among females (3%). After calculating all those risks, 79% of males and 60% of females practiced any risky behaviors at their sexual debut resulting in a two times higher likelihood of overall risky sexual debut among males (*p*-value = 0.003).

**Table 3:** Comparison of risky sexual behaviors at sexual debut between male and female youths

	**Males (n = 100)**	**Females (n = 135)**	**P value**
**Risky behavior at sexual debut**	**n (%)**	**n (%)**	
Early age (age ≤ 16)	34 (34.0)	46 (34.1)	1
Premarital sex	35 (35.0)	6 (4.4)	< 0.001
Condoms/contraceptives non-use	52 (52.0)	64 (47.4)	0.5
Non-regular partner	27 (27.0)	4 (3.0)	< 0.001
**Risky sexual debut (any of above behaviors)**	**79 (79)**	**81 (60)**	**0.003**

**Figure 2:**
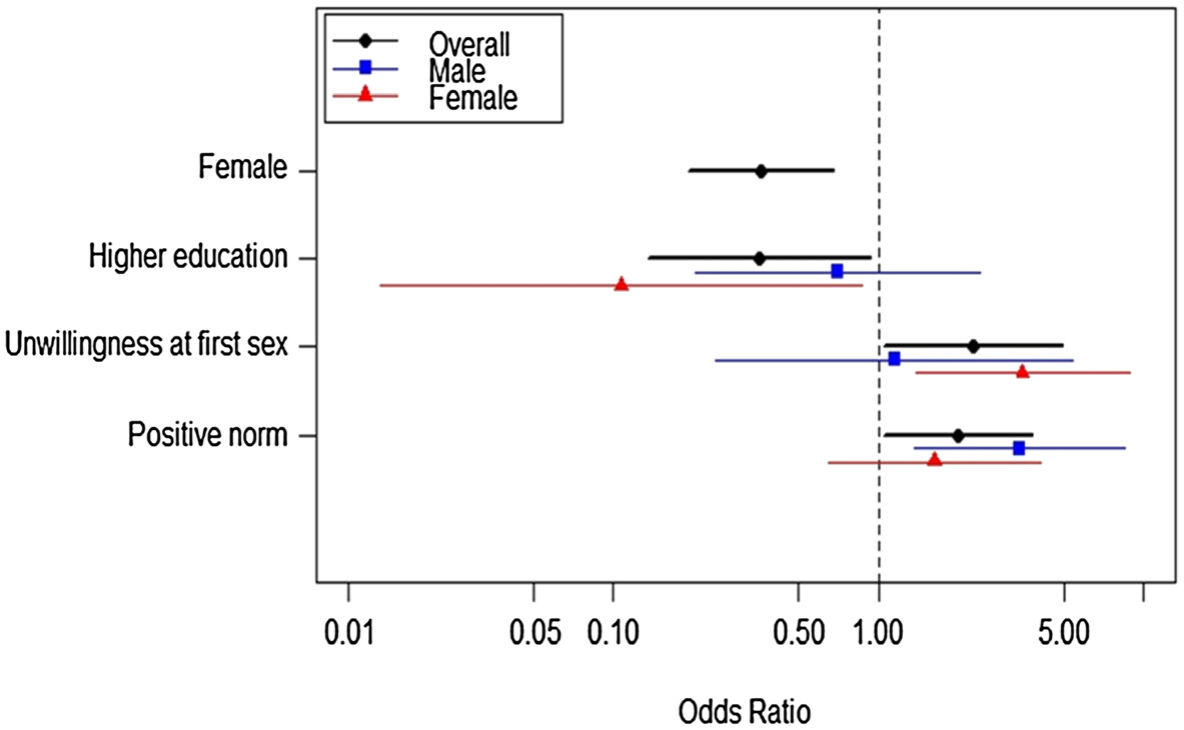
Overall and sex stratified factors associated with risky sexual debut.

Figure 2 compares factors associated with risky sexual debut from the multivariate analysis. Among all youths, being female and having a higher level of education were significant protective factors of risky sexual debut while an unwillingness to have sex at sexual debut and positive norms of premarital sex were significant risk factors. After separate analysis, only unwillingness to have sex at sexual debut increased the risk of risky sexual debut among females while that variable didn’t affect male’s risky sexual debut. While having a high school education decreased the risk among females, education was not associated with males’ risky sexual debut. Having a positive norm of premarital sex was the only risk factor that increased the likelihood of risky sexual debut among males though this factor had no effect on female’s risky sexual debut.

## Discussion

The levels of exposure to SRH information and risky sexual debut among poor youths in suburban communities of Mandalay City, Myanmar were unfavorable. Gender differences were clearly seen for both exposure to SRH information and risky sexual debut. Specific influential factors affected RH behaviors differently between males and females. While parental guardianship and media exposure influenced exposure to SRH information among males, these factors were not significant among females. Education and willingness to have sex were influential for females’ exposure to risky sexual debut; however, only having a positive norm of premarital sex affected males’ exposure to risky sexual debut.

The level of exposure to SRH information (mainly on sexuality and STI/HIV) among males and females in our study was lower compared to that of Indian and African studies conducted among youths [20-22]. On the other hand, the level of early sexual debut among both males and females was alarmingly high in our study compared to a Chinese study in which sexual debut before age 18 among youths was rare [10]. The rate of premarital sex in our study was higher than that in studies conducted in Nigeria and Eastern Ethiopia; however the rate of condom non-use at sexual debut and having sex with a non-regular partner was similar to our study [11,23]. The possible explanation for these differences can be due to the different study populations and settings. The participants in the other studies were mostly drawn from the general population [23] or university students [11] whereas our study was conducted among poor suburban youths who were socially and economically vulnerable.

Among known socio-economic variations affecting youth’s RH behaviors, gender played a significant role in many studies in different settings as well as in our study [5,9,10]. Our study found that females had obvious socio-economic vulnerabilities compared to males, e.g. having a lower income and education, being unemployed, and having a higher exposure to sex and marriage. It is notable that the rate of unwillingness at first sex or being forced into sex was extremely high among females in our study compared to a study conducted among out-of-school youths in China [24]. These findings confirmed the existence of gender inequality among youths in our study area.

Despite those limited socio-economic conditions, females had a higher exposure to SRH information, especially on STI/HIV compared to males. A similar finding was seen in a study conducted among African American adolescents, in which females had a higher knowledge of STI/HIV [25]. In that study, sexual experiences played a major role to get a higher knowledge of STI/HIV; however, youth’s preferred sources of information might be the main reason of this difference in our study. We found that females received SRH information mainly from health personnel but most males responded that they received SRH information from unreliable sources, such as friends or older youths in their community. This finding was also evident in a study among Indian male youths in which participants happened to receive SRH information from unreliable sources [26].

In addition to gender, we also found that age and schooling status influenced the exposure to SRH information among both males and females. Adolescents had lower exposure to SRH information compared to older youths probably because of their own embarrassment to access the available information or the cultural barrier which deters them to do so [27-29]. Out-of-school youths had a lower exposure to SRH information, a finding similar to a study conducted in China [24]. Even though the importance of schooling status for SRH education has been internationally recognized and addressed, this finding highlights the lack of solutions on providing SRH information to this vulnerable out-of-school group in recent SRH education programmes.

It is notable that exposure to mass media and parental guardianship also affected the exposure of SRH information only among males. A study conducted among young-unmarried men in India showed that these men also received SRH information mainly from the mass media [26]. In contrast, mass media was significantly associated with beliefs that increased the likelihood of sexual exposure among adolescents in the US [30], a finding which highlights the importance of mass media among male youths and its ambiguous effects on their RH behaviors. On the other hand, in our study having both parents as guardians was a positive influential factor on males’ exposure to SRH information. Thus, parental support or parental control was important not only for young people’s SRH behaviors, but also for their exposure to SRH information [24,31].

Gender differences were also seen in risky sexual debut behaviors among the study participants. Females had a lower odds of having a risky sexual debut behaviors compared to males, a finding supported by many other Asian and African studies [5,9-11]. This might probably be because males have a higher interest in sexual pleasure, freedom of motivations to have sex, and lesser feelings of guilt and shame after sex compared to females [9,32]. In addition to gender, education, willingness to have sex at sexual debut and positive norms of premarital sex were important for youths’ exposure to risky sexual debut.

A higher education level was a protective factor of risky sexual debut among females, a finding which was supported by many other studies [33-35]. Education is an essential enabling factor which improves all aspects of women’s RH life. This finding could probably draw the policy makers’ attention to the importance of young girls’ school enrolment in these poor communities. The finding that females who had sex unwillingly or were forced into sex at their sexual debut were more likely to be exposed to risky sexual behaviors was also supported by a systematic review conducted in the US [36]. Women with violent or controlling male partners were at increased risk of HIV infection and risky sexual behaviors. The postulation was that abusive men were more likely to have HIV and impose risky sexual practices on their partners [37].

Males having a positive norm of premarital sex (males who replied that their friends had premarital sex, that it was very common and usual in their community and those who perceived that there was nothing wrong with premarital sex) were more likely to be exposed to risky sexual debut in our study. Sexual norms are important among youths. While positive norms of premarital sex increased the risk of risky sexual debut, virginity norms of males delayed sexual debut among rural Ethiopian youths [38]. This confirms the fact that complexity of social norms affects youth’s sexual behaviors differently through peers or society [39].

Some strengths and limitations of our study should be mentioned. One of the strengths of this study is that it elicited gender differences in exposure to SRH information and risky sexual debut among youths in hard-to-reach, poor, suburban communities of Myanmar. However, there were some limitations. First, the issue of risky sexual debut is culturally sensitive; therefore, some youths might have been reluctant to reveal their sexual behavior in detail. This may have caused an underreporting in the prevalence of this outcome. However, anonymity and confidentiality were emphasized and the interviewers were of the same gender as the youths they interviewed. Second, the youths in our study were located in poor communities, thus the generalizability of our findings may be limited. Third, even though considerable efforts were undertaken by the community leaders and municipal authorities to ensure a complete listing of youths in the study area, some youths may have not been included in the sampling frame.

## Conclusions

Gender was significantly associated with exposure to SRH information and risky sexual debut among youths. These gender differences in sexual behaviors and risks highlight the need for gender specific solutions and approaches to achieve better SRH outcomes among youths. Policy makers as well as local RH care providers should be aware of those different influential factors between males and females. The reliability and sufficiency of current SRH information available in mass media should be assessed and enhanced accordingly. More dissemination of appropriate SRH information via youth-accessible mass media (e.g. television, radio) should be carried out, especially among males. Female empowerment should be emphasized (e.g. education, employment) in these poor communities while male norms on premarital sex should be corrected accordingly through intervention programs. RH risks among this vulnerable group need to be continuously monitored.

## Abbreviations

SRH: Sexual and reproductive health.

## Competing interests

The authors declare that there are no competing interests.

## Authors’ contributions

PPTZ designed the study, conducted the data collection process, analyzed and interpreted the data, and prepared the manuscript. TL provided supervision on all aspects of the study and manuscript preparation. EM helped with data management, statistical analysis and manuscript preparation. TTH helped to conceptualize the study, supported the data collection process and commented on the manuscript. All authors read and approved the final manuscript.

## Pre-publication history

The pre-publication history for this paper can be accessed here:

http://www.biomedcentral.com/1471-2458/13/1122/prepub
